# Patient-tailored modulation of the immune system may revolutionize future lung cancer treatment

**DOI:** 10.1186/1471-2407-12-580

**Published:** 2012-12-05

**Authors:** Marlies E Heuvers, Joachim G Aerts, Robin Cornelissen, Harry Groen, Henk C Hoogsteden, Joost P Hegmans

**Affiliations:** 1 Department of Pulmonary Medicine, Erasmus Medical Center, Postbox 2040, 3000 CA, Rotterdam, The Netherlands; 2 Department of Pulmonary Medicine, Amphia Hospital, Breda, The Netherlands; 3 Department of Pulmonary Medicine, University Medical Centrum Groningen, Groningen, The Netherlands

**Keywords:** Lung cancer, Tumor microenvironment, Immune system, Personalized medicine, Cancer immunology

## Abstract

Cancer research has devoted most of its energy over the past decades on unraveling the control mechanisms within tumor cells that govern its behavior. From this we know that the onset of cancer is the result of cumulative genetic mutations and epigenetic alterations in tumor cells leading to an unregulated cell cycle, unlimited replicative potential and the possibility for tissue invasion and metastasis. Until recently it was often thought that tumors are more or less undetected or tolerated by the patient’s immune system causing the neoplastic cells to divide and spread without resistance. However, it is without any doubt that the tumor environment contains a wide variety of recruited host immune cells. These tumor infiltrating immune cells influence anti-tumor responses in opposing ways and emerges as a critical regulator of tumor growth. Here we provide a summary of the relevant immunological cell types and their complex and dynamic roles within an established tumor microenvironment. For this, we focus on both the systemic compartment as well as the local presence within the tumor microenvironment of late-stage non-small cell lung cancer (NSCLC), admitting that this multifaceted cellular composition will be different from earlier stages of the disease, between NSCLC patients. Understanding the paradoxical role that the immune system plays in cancer and increasing options for their modulation may alter the odds in favor of a more effective anti-tumor immune response. We predict that the future standard of care of lung cancer will involve patient-tailor-made combination therapies that associate (traditional) chemotherapeutic drugs and biologicals with immune modulating agents and in this way complement the therapeutic armamentarium for this disease.

## Review

### Current NSCLC treatment

Treatment of lung cancer is currently based on the patient’s clinical signs and symptoms, tumor stage and subtype, medical and family history, and data from imaging and laboratory evaluation. Most conventional cancer therapies, such as radiotherapy and chemotherapy are restricted by adverse effects on normal tissue. Currently NSCLC therapy is moving towards personalized medicine where the genetic profile of each patient’s tumor is identified and specific therapies that inhibit the key targets of the oncogenic activation are targeted. In approximately 60% of all NSCLC cases, specific mutations can be identified, of which ± 20% can be targeted with specific drugs at this moment (e.g. erlotinib, gefitinib, crizotinib). However, most patients receiving conventional cancer treatments or targeted drugs will experience a relapse of tumor growth at a certain time. This sobering outcome demonstrates the necessity of innovative approaches in NSCLC treatment.

Recently, experimental findings and clinical observations have led to cancer-related immune inflammation being acknowledged as an additional hallmark of cancer [1,2]. There is currently overwhelming evidence that several immunological cell types of the host influence cancer incidence, cancer growth, response to therapy and thereby the prognosis of the disease. However, the immune system plays a paradoxical role by either preventing cancer growth or in sculpting tumor escape and stimulates its development. A better understanding of the interaction between cancer cells and host immune cells within the tumor environment is of importance for further progress in cancer treatment. This is an extremely difficult task because of the complicated cancer-host immune interactions. The field that studies these interactions, termed cancer immunology, is rapidly progressing. It provides insights into the contribution of the immune system in processes such as tumor invasiveness, metastasis, and angiogenesis and may predict the response to treatment. Most importantly, it also provides opportunities for improved anti-cancer therapies. Modulation of the patient’s immune system combined with anti-tumor treatments offers the prospect of tailoring treatments much more precisely and better efficacy for patients with advanced lung cancer.

### Immune cells involved in tumorogenesis

**Figure 1:**
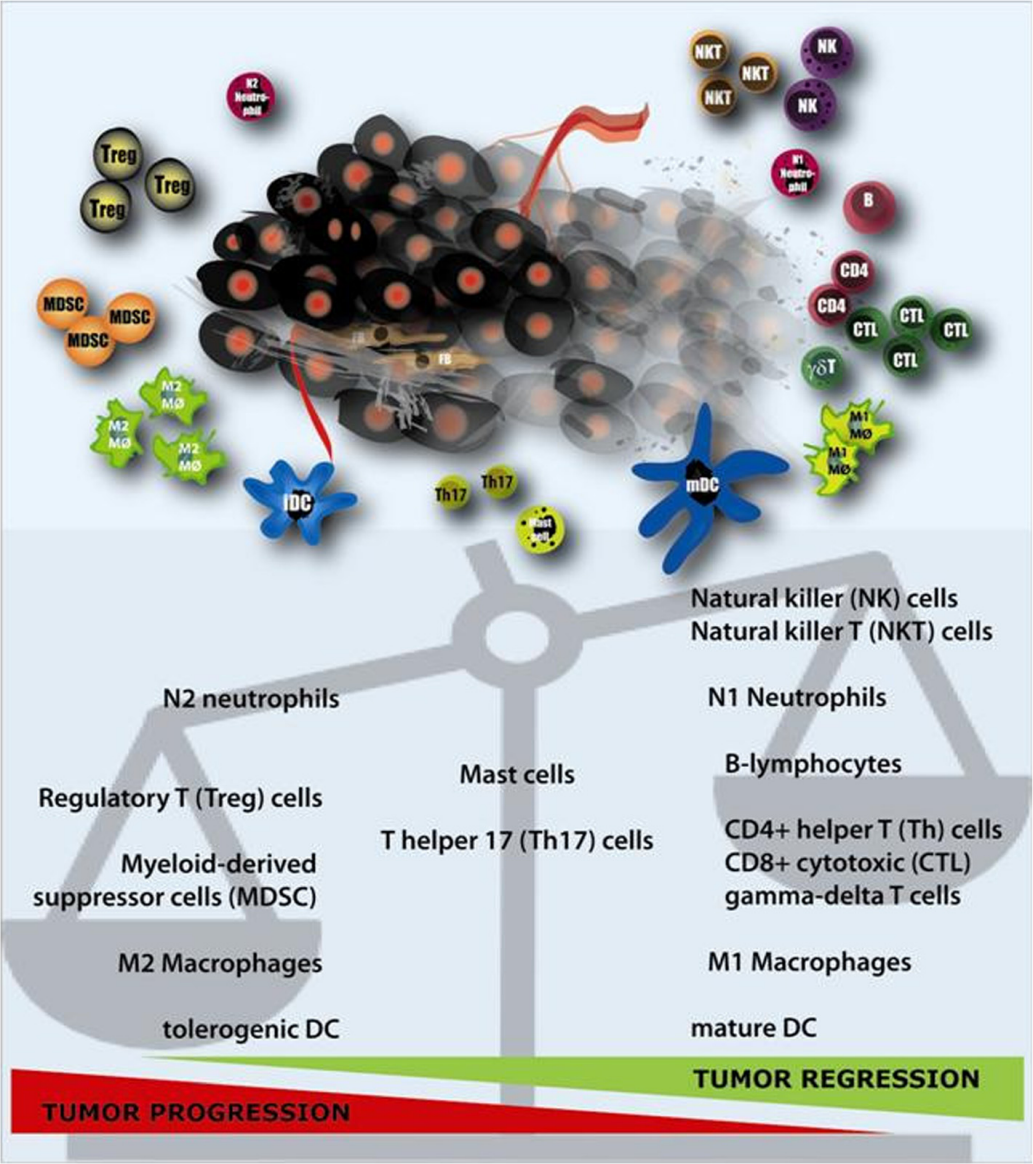
**The tumor microenvironment is a heterogeneous and complex system of tumor cells (black) and ‘normal’ stromal cells, including endothelial cells and their precursors, pericytes, smooth-muscle cells, and fibroblasts of various phenotypes, located within the connective tissue or extra-cellular matrix (e.g. collagen). **Leukocyte infiltration is an important characteristic of cancer and the main components of these infiltrates include natural killer (T) cells, neutrophils, B- and T-lymphocyte subsets, myeloid derived suppressor cells, macrophages and dendritic cells [3-7]. Based on their functions, these cells can be divided into cells with a potentially positive impact on the antitumor response (right) and cells with a detrimental effect (left). From mast cells and T helper 17 cells it is yet ambiguous what kind of effect these cells have within the micro-environment. The net effect of the interactions between these various cell types and their secreted products within the environment of an established tumor participates in determining anti-tumor immunity, angiogenesis, metastasis, overall cancer cell survival and proliferation.

The individual immune related tumor infiltrating cell types are discussed below (Figure 1).

#### Natural killer (T) cells

Natural killer (NK) cells (expressing the surface markers CD16 and CD56, but not CD3) are lymphocytes that play an important role in the rejection of tumors without previous sensitization and without restriction by the major histocompatibility complex (MHC) [8,9]. NK cells eradicate tumors through multiple killing pathways, including direct tumor cell killing. They also secrete cytokines and chemokines like Interleukin (IL) IL-10, Tumor Necrosis Factor (TNF)-α, and the principal NK-derived cytokine Interferon (IFN)-γ, which can coordinate the innate and adaptive immune responses to tumor cells and may lead to apoptosis of the attacked cells.

A large cohort study showed that an increase in NK cells in tumor tissue is a strong independent prognostic factor for the survival of lung cancer patients [10]. This is confirmed in mouse models, showing that stimulation of NK cell function protected against NSCLC metastasis [11,12], while depletion enhanced lung cancer metastasis [13]. However, it was recently shown that although the frequencies of NK cells in blood do not differ from healthy controls, stimulated blood NK cells from NSCLC patients with advanced disease had a reduced granzyme B and perforin A expression, lower production of IFN-γ, and decreased cytotoxic function indicating that these cells are functionally impaired in comparison with healthy controls [14,15]. Adoptive transfer of allogeneic, *in vitro* activated and expanded NK cells from haploidentical donors was proven potentially clinically effective in NSCLC [16].

Natural killer T (NKT) cells (CD16^+^, CD56^+^, CD3^+^) are a subset of NK cells that have been found in the peripheral blood, tumor tissue and pleural effusions of lung cancer patients in decreased numbers and with reduced functions [17,18]. It has been shown that NKT cells in cancer patients produce a decreased amount of IFN-γ and are therefore less effective than NKT cells in healthy controls [19,20]. They are currently exploited for cancer treatment by harnessing these cells with CD1d agonist ligands [21,22], or by adoptive transfer of NKT cells activated *in vitro*[23].

#### Mast cells

Accumulation of mast cells is common in angiogenesis-dependent conditions, like cancer, as mast cells are a major provider of proangiogenic molecules vascular endothelial growth factor (VEGF), IL-8, transforming growth factor (TGF)-β [24]. The density of mast cells in NSCLC tumors is correlated with microvessel density [25] and mast cells / histamine has a direct growth promoting effect on NSCLC cell lines *in vitro*[26]. However, the role of mast cells in the prognosis in NSCLC remains controversial [25,27-29]. Tumor-infiltrating mast cells can directly influence proliferation and invasion of tumors, by histamine, IL-8 and VEGF while the production of TNF-α and heparin can suppress tumor growth [26,30]. It has been shown that in NSCLC mast cell counts were noted to increase as tumor stage increased while another study did not show this correlation [24,29]. Mast cells also play a central role in the control of innate and adaptive immunity by interacting with B and T cells (in particular Treg) and dendritic cells. The controversy of mast cells in cancer seems to be related to the type, microenvironment and stage of cancer and their role may depend on the tumor environment [29,31,32]. Therapeutic intervention by targeting mast cells, although technically possible [33], is too early without more knowledge on the paradoxical role of these cells in individual cases.

#### Neutrophils

Neutrophils play a major role in cancer biology. They make up a significant portion of the infiltrating immune cells in the tumor and the absolute neutrophils count and the neutrophils to lymphocyte ratio in blood are independent prognostic factors for survival of NSCLC [34-36]. Neutrophils are attracted to the tumor under the influence of specific chemokines, cytokines and cell adhesion molecules. Tumor-associated neutrophils (TAN) have polarized functions and can be divided into the N1 and N2 phenotype in a context-dependent manner [37,38]. The N1 phenotype inhibits tumor growth by potentiating T cell responses while the N2 phenotype promotes tumor growth [3]. The antitumor activities of N1 neutrophils include expression of immune activating cytokines (TNF-α, IL-12, GM-CSF, and VEGF), T cell attracting chemokines (CCL3, CXCL9, CXCL10), lower expression of arginase, and a better capacity of killing tumor cells *in vitro*. N2 neutrophils support tumor growth by producing angiogenic factors and matrix-degrading enzymes, support the acquisition of a metastatic phenotype, and suppress the anti-tumor immune response by inducible nitric oxide synthase and arginase expression. Neutrophils also influence adaptive immunity by interacting with T cells [39], B-cells [40], and DC [41]. In resectable NSCLC patients, intratumoral neutrophils were elevated in 50% of the patients and this was associated with a high cumulative incidence of relapse [42]. Recently, Fridlender *et al.* showed that TGF-β acquired the polarized N2 tumor promoting phenotype of neutrophils in a murine lung cancer model, and blocking of TGF-β shifted towards N1 tumor rejecting neutrophils with acquisition of anti-tumor activity *in vitro* and *in vivo*[43]. Blockade of TGF-β in humans might be a potential utility to prevent polarization towards the protumorigenic N2 phenotype and thereby may result in retarding tumor growth.

#### B lymphocytes

B-cells may affect the prognosis of patients with lung cancer, as patients with stage I NSCLC contain more intratumoral germinal centers with B-lymphocytes than patients with stages II to IV [44]. These tertiary (T-BALT) structures provide some evidence of an adaptive immune response that could limit tumor progression in some patients. For instance, the production of antibodies by B-cells can activate tumor cell killing by NK cells and other inflammatory cells [45]. Auto-antibodies against tumor antigens are commonly found in patients with lung cancer [46-48] and can inhibit micrometastasis [49]. Recently, it has been shown in mice that antibodies produced by B cells interact with and activate Fcγ receptors on macrophages and in this way orchestrate antitumor activity [50] or tumor-associated macrophages (TAM)-mediated enhancement of carcinogenesis [51]. Thus, the role of B cells seems depending on the context.

#### CD4+ and CD8+ lymphocytes

CD4+ cells and CD8+ cells represent the strong effectors of the adaptive immune response against cancer [52]. There is controversy on the impact of T cells and their localization on the prognosis of lung cancer [53-59]. This may be caused by the presence of a special subset of T cells, the regulatory T cells, and myeloid-derived suppressor cells which are discussed below. Also tumor-derived factors can exhaust T lymphocytes or induce their apoptosis [60]. Recently it has been shown that cytotoxic T lymphocytes (CTL) within the tumor (the tumor-infiltrating lymphocytes [TIL]) are of beneficial prognostic influence in resected NSCLC patients in both adenocarcinoma [61] and squamous cell carcinoma [62]. Tumor-specific CD8+ effector T-cells are normally present at a low frequency in cancer patients, but can be expanded up to 50% of the total circulating CD8+ T-cells by dendritic cell vaccination or adoptive T-cell transfer therapy [63-65]. To enhance existing anti-tumor responses, recombinant CD40 ligand or CD40 activating antibodies are investigated as substitute for CD4+ T cell help [66]. Blocking T cell inhibitory molecules such as cytotoxic T lymphocyte antigen-4 (CTLA-4), lymphocyte activation gene-3 (LAG-3), T cell immunoglobulin mucin-3 (TIM-3), and programmed death-1 (PD-1) are currently investigated in NSCLC to improve T cell homing and effector functions [67,68]. Successes of these experimental therapies in small subsets of patients demonstrate that CTL can be directed against the tumor but mechanisms to induce CTL or overcome the inactivation of T cell function seems necessary to enable more patients from these treatments.

#### Regulatory T cells

Regulatory T cells (Treg), characterized by CD4^+^, CD25^+^, Foxp3^+^, and CD127^-^, are T lymphocytes that are generated in the thymus (natural Treg) or induced in the periphery (induced Treg) when triggered by suboptimal antigen stimulation and stimulation with TGF-β and IL-10 [69]. Treg are further characterized by the expression of glucocorticoid-induced TNF-receptor-related-protein (GITR), lymphocyte activation gene-3 (LAG-3), and cytotoxic T-lymphocyte-associated antigen 4 (CTLA4).

In cancer patients, Treg confer growth and metastatic advantages by inhibiting anti-tumor immunity. They have this pro-tumoral effect by promoting tolerance via direct suppressive functions on activated T-cells or via the secretion of immunosuppressive cytokines such as IL-10 and TGF-β [70,71]. Treg are present in tumor tissue [72,73] and increased in peripheral blood of NSCLC patients compared to healthy controls [74,75]. This increase in Treg was found to promote tumor growth and was correlated with lymph node metastasis [56,73,76,77] and poor prognosis [73,78]. Many factors can increase Treg in NSCLC tumors, among them are thymic stromal lymphopoietin (TSLP) [79] and intratumoral cyclooxygenase-2 (COX-2) expression [80]. Treg are considered the most powerful inhibitors of antitumor immunity [81]. As a result, there is substantial interest for overcoming this barrier to enhance the efficacy of cancer immunotherapy. Strategies include I). Treg depletion by chemical or radiation lymphoablation or using monoclonal antibodies or ligand-directed toxins (daclizumab, basiliximab, denileukin diftitox [Ontak^TM^, RFT5-SMPT-dgA, and LMB-2) or with metronomic cyclophosphamide. II). Suppression of their function (ipilimumab, tremelimumad [anti-CTLA4], DTA-1 [anti-GITR], denosumab [anti-RankL], modulation of Toll-like receptor, OX40 stimulation or inhibiting ATP hydrolysis using ectonucleotidase inhibitors). III). Inhibition of tumoral homing by blocking the selective recruitment and retention of Treg at tumor sites, e.g. CCL22, CXCR4, CD103, and CCR2. IV). Exploitation of T-cell plasticity by modulating IL-6, TGF-β, and PGE2 expression, e.g. the COX-2 inhibitor celecoxib [82]. Till now, a strategy that specifically target only Treg and no effector T cells is lacking and procedures that depletes or modulates all Treg should be avoided to minimize the risk of autoimmune manifestations. However, studies modulating Treg in patients are providing some early encouraging results supporting the concept that Treg inhibitory strategies have clinical potential, particularly in those therapies that simultaneously stimulate antitumor immune effector cells.

#### Gamma delta T cells

Human γδ-T cells constitute 2-10% of T cells in blood and exhibit natural cytolytic activity in an MHC-unrestricted manner for microbial pathogens and tumor cells. A special TCR on γδ-T cells recognizes small nonpeptide antigens with a phosphate residue and isopentenylpyrophosphate (IPP) that accumulate in tumor cells [83]. Because γδ-T cells recognize target cells in a unrestricted manner, they may exert antitumor effects even on tumor cells with reduced or absent expression of HLA and/or tumor antigens or by provision of an early source of IFN-γ [83,84]. Phase I clinical trials of *in vivo* activation of γδ-T cells with zoledronic acid plus IL-2 or adoptive transfer of *in vitro* expanded γδ-T cells are being conducted at present for lung cancer [85-87].

#### Th17 cells

Th17 cells are a subpopulation of CD4^+^ T helper cells that are characterized by the production of interleukin-17 (IL-17, also known as IL-17A). IL17 plays an important role in the host defenses against bacterial and fungal infections by the activation, recruitment, and migration of neutrophils [88,89]. *In vitro* experiments have shown that IL-1β, IL-6, and IL23 promote Th17 generation and differentiation from naïve CD4^+^ T cells [90]. Among the other cytokines secreted by Th17 cells are IL-17F, IL-21, IL-22, and TNF-α. The role of Th17 cells in cancer is poorly understood. Th17 cells accumulate in malignant pleural effusion from patients with lung cancer [90]. Also higher levels of IL-17A were detected in serum and in tumor lesions of lung adenocarcinoma patients, indicating a potential role of these cells in cancer [91]. It has been shown that Th17 cells encouraged tumor growth by inducing tumor vascularization or enhancing inflammation, but other studies revealed also opposite roles for Th17 cells. Recent data indicate that IL-17 may play a role in the metastasis of lung cancer by promoting lymphangiogenesis and is therefore an independent prognostic factor in both overall and disease-free survival in NSCLC [92]. However, there is a distinct role for Th17 and Th17-stimulated cytotoxic T-cells in the induction of preventive and therapeutic antitumor immunity in mice by the promoted recruitment of several inflammatory leukocytes, like DC, CD4^+^ and CD8^+^ cells [93]. So, it is controversial whether Th17 cells in cancer are beneficial or antagonistic; this may be dependent on the tumor immunogenicity, the stage of disease, and the impact of inflammation and angiogenesis on tumor pathogenesis [94].

#### Myeloid-derived suppressor cells

Myeloid-derived suppressor cells (MDSC) are a heterogeneous population of immature myeloid cells and myeloid progenitor cells. MDSC inhibit T cells activation [95,96] in a nonspecific or antigen-specific manner, alter the peptide presenting ability of MHC class I molecules on tumor cells [97], influence B-cells [98], block NK cell cytotoxicity [99-101], inhibit dendritic cell differentiation [102], and expand Treg [103,104] signifying their crucial contribution in constituting a tumor suppressive environment. Furthermore, there is compelling evidence that MDSC, by secreting MMP9 and TGF-β1, are also involved in angiogenesis, vasculogenesis, and metastatic spread [105].

MDSC suppress the immune system by the production of reactive oxygen species (ROS), nitric oxide (NO), peroxynitrite and secretion of the cytokines IL-10 and TGF-β [106]. Upregulated arginase-I activity by MDSC depletes the essential amino acid L-arginine, contributing to the induction of T cell tolerance by the down regulation of the CD3ζ chain expression of the T cell receptor [107-110]. However, the mechanisms that are used to suppress the immune responses are highly dependent on the context of the microenvironment [111].

An increased subpopulation of MDSC in the peripheral blood of NSCLC patients was detected that decreased in those patients that responded to chemotherapy and patient undergoing surgery [112]. Because MDSC play an important role in mediating immunosuppression, they represent a significant hurdle to successful immune therapy in NSCLC. Therefore, targeting MDSC *in vivo* with drugs like 5-fluorouracil (5FU), gemcitabine or VEGF / c-kit blockers (e.g. sunitinib, imatinib, dasatinib) to elicit more potent anticancer effects is an exciting development [113-115]. Treatment of mice with all-trans retinoic acid (ATRA), along with NKT help, convert the poorly immunogenic MDSC into fully efficient APC and in this way reinforced anti-tumor immune responses [116]. Other MDSC suppressing or differentiation-inducing agents recently reported are 5-aza-2′-deoxycytidine, curcumin, IL-10, anti-IL4R aptamer, and vitamin D3 [117-120]. Agents that decrease arginase activity, ROS and/or iNOS expression by MDSC include Nor-NOHA, 1-NMMA, cyclooxygenase 2 inhibitors (celecoxib [121]), phosphodiesterase 5 inhibitors (sildenafil, tadalafil [122]) or reactive oxygen species inhibitors (nitroaspirin [123]). These agents promise to be a fruitful avenue of investigation in the coming years to overcome immune suppression associated by MDSC in advanced tumors [113,114].

#### Tumor–associated macrophages

Macrophages are part of the innate immune system and play important roles in the first line of defense against foreign pathogens. They can be divided into M1 macrophages (classical activation) and M2 macrophages (alternative activation). M1 macrophages attract and activate cells of the adaptive immune system and have anti-tumor and tissue destructive activity, while the M2 phenotype has been linked to tumor-promoting activities by subversion of adaptive immunity, promoting tumor angiogenesis and supporting cancer cell survival, proliferation, invasion and tumor dissemination. Macrophages in tumors are usually referred to as tumor-associated macrophages (TAM) and their presence can be substantial (10–65% of the tumor stroma). In the beginning, the TAM mainly consist of M1-like macrophages however, when the tumor starts to invade and vascularize, there is a skewing towards the M2 phenotype [124,125]. This takes place especially at those regions in the tumor that are hypoxic [126].

It has been reported by several groups that there is an association between the number of tumor islet macrophages and NSCLC survival [58,127-132]. Moreover, when looking at the different phenotypes of TAM (M1 and M2), it is shown that high numbers of M1 macrophages infiltrating the tumor are correlated with improved survival [130,133]. On the other hand, the presence of M2-like macrophages is associated with poor clinical outcome [130,133].

Several strategies are currently investigated that influence M2 macrophages at multiple levels. For example, blockade of factors and cytokines secreted by tumor or immune cells to limit the induction of M2 macrophages are investigated [134-136], however this results in loss of typical M2 markers but not their function [137]. It has been shown that inhibiting IκB kinase (IKK) reprograms the M2 phenotype to the M1 subset [138,139]. Also CD40 therapy seems to skew tumor-infiltrating macrophages towards the M1 phenotype [140]. Influencing the attraction, the polarization or the activation of M2 macrophages may improve survival when combined with standard or other immunotherapeutic regimens.

#### Dendritic cells

Dendritic cells (DC) are widely acknowledged as the central surveillance cell type and play an important role in the activation of lymphocyte subsets to control or eliminate human tumors. Upon encountering tumor cells or tumor-associated antigens, DC engulf this material and begin migrating via lymphatic vessels to regional lymphoid organs. The density immature DC (Langerhans cell and interstitial DC) and mature DC, present in the tumor microenvironment is highly predictive of disease-specific survival in early-stage NSCLC patients [141] and the presence of DC in resected NSCLC material is a good prognostic factor [10,142]. Interaction between the DC and tumor cells results in the release of antitumour cytokines [143,144]. This suggests that DC within the tumor microenvironment of early-stage NSCLC are capable in initiating adaptive immune responses in situ [145-147].

In the peripheral blood and regional lymph nodes of lung cancer patients, the number and function of mature DC is dramatically reduced [148,149], partly due to abnormal differentiation of myeloid cells (e.g. MDSC) [150]. Tumor cells, stromal cells like fibroblasts, and tumor-infiltrating immune cells and/or their secreted products, like VEGF, M-CSF, IL-6, IL-10, and TGF-β are also responsible for systemic and local DC defects [151-154]. Affected DC are impaired in their ability to phagocytose antigen and to stimulate T cells, leading to a defective induction of anti-tumor responses.

NSCLC-derived DC produce high amounts of the immunosuppressive cytokines IL-10 and TGF-β [155]. It has been shown that the T cell co-inhibitory molecule B7-H3 and programmed death receptor-ligand-1 (PD-L1) are upregulated on tumor residing DC and these molecules conveys mainly suppressive signals by inhibiting cytokine production and T cell proliferation [156,157].

Tumor-induced modulation is one of the main factors responsible for tumor immune escape and correction of DC function might be a requirement to develop more effective immunotherapeutic strategies against cancer. This might include targeting of those factors with neutralizing antibodies (e.g. anti-VEGF, anti-IL-6) to revert some of the inhibitory effects on DC. Another interesting finding is that culturing monocytes from cancer patients *ex vivo*, to circumvent the suppressive activity of the tumor milieu, generates DC with a capacity to stimulate allogeneic T cells [158,159]. [160] This finding is important for active DC-based immunotherapeutic approaches, where DC are generated *ex vivo* from monocytes and after arming with tumor-associated antigens, reinjected into the patient with the intension to restore proper presentation of tumor associated antigens (TAA) and T cell activation [161-163]. This concept is currently tested for NSCLC in therapeutic reality with encouraging results on the immune response, safety and tolerability, despite the small sample sizes of the trials [161-163].

#### Immunogenic cell death biomarkers

Lung cancer is a complex disease with limited treatment options, mainly caused by the close relationship between neoplastic cells and healthy cells. To develop a more effective treatment for lung cancer, we have to focus on the complex interactions that tumor cells have with the local stromal compartment and the involved immune cells, and all of their secreted factors. There is growing evidence that the efficacy of many traditional therapeutic treatments depends on their ability to induce proper immunogenic tumor cell death. This specific release of signals upon tumor cell death may lead to immune activation, and in particular anti-tumor immunity, that contribute to the therapeutic outcome for patients [164,165].

There are different candidate immune biomarkers that can predict the efficacy of specific NSCLC anticancer therapies [166,167]. In NSCLC, nucleosomes have already been proven useful for the early estimation of response to chemotherapy [168-170]. Presence of mature dendritic cells and CD4+ or CD8+ lymphocytes in NSCLC tumors are independent prognostic factors for overall survival, as described above [55,59,171,172]. In addition, other potentially pivotal markers for lung cancer are p53-specific autoantibodies and pyridoxal kinase (PDXK), the enzyme that generates the bioactive form of vitamin B6 [173]. Also a group of immunogenic cell death biomarkers called damage-associated molecular pattern (DAMP) molecules, can serve as prognostic markers for response to therapy and prognosis in cancer patients [174]. DAMPs, such as surface-exposed calreticulin (ecto-CRT) and the high-mobility group box 1 protein (HMGB1); are released in the blood circulation by late apoptotic and necrotic cells upon oxidative and endoplasmic reticulum (ER) stress. In peripheral blood, they bind to specific immune cells and trigger protective T cell responses and promote phagocytosis. One of the main functions of HMGB1 is the binding to specific receptors on dendritic cells and other antigen presenting cells, such as receptors for advanced glycation endproducts (RAGE) and toll-like receptors 4 (TLR4). It has been described that the release of DAMP during cell death is essential for the sustained therapy response after chemotherapy and the efficiency of HMGB1 was found to be increased when bacterial lipopolysaccharide (LPS), DNA or nucleosomes were bound to it. Knockdown of HMGB1 was observed to be associated with reduced anticancer immune response and poor therapy outcome. In contrary, overexpression of HMGB1 and its receptor RAGE is pivotal for the metastasizing of the tumor cells as it promotes neoangiogenesis [175]. Markers of immunogenic cell death are becoming a valuable tool in clinical practice for diagnosis and prediction of response to NSCLC therapy and prognosis [167].

Next to DAMP, there are other approaches using RNA- and DNA-based immune modifiers to augment cancer therapy efficacy by stimulating the immune system. Bacterial DNA is immunostimulatory and can be replaced using synthetic oligodeoxynucleotides (ODN), for instance CpG oligodeoxynucleotides. CpG ODN are synthetic DNA sequences containing unmethylated cytosine-guanine motifs with potent immune modulatory effects via TLR 9 on DC and B cells [176]. They can induce cytokines, activate NK cells, and elicit T cell responses that lead to strong antitumor effects. It has been shown that CpG ODN downregulates regulatory T cells and TGF-β in peripheral blood of NSCLC patients [177].

Overall, analysis of new and conventional therapeutic strategies should not only be focused on the direct cytotoxic effects of tumor cells but also on the initiation of proper immune responses. Simultaneous modulation of the immune system by immune therapeutic approaches can then induce synergistic anticancer efficacy [178]. Overall, the composition of the immunological cells and cell death markers in the host is, next to the mutation analysis and histological features of the tumor, likely to determine the response to specific chemotherapeutic agents and the prognosis of the patients.

## Conclusion

In this review, we have shown that the immune system plays a dual role in cancer development and progression and determines the response to treatment in NSCLC. These complex interactions between diverse immune cell types and tumor cells that can actively favor tumor rejection as well as tumor progression, depends on the tumor type, stage and the types of immune cells that are involved. The data presented here reinforce the importance of full understanding of the intricacy of the cellular interactions within the tumor microenvironment. There is a rapid progress in the field of the cancer immunology and the development of novel cancer immunotherapy approaches. Therefore, tumor immunology will probably be used more commonly in clinical practice in the future, as increasing evidence indicates that the effectiveness of several chemotherapies depends on the active contribution of the different immune effectors. Selecting conventional chemotherapeutic agents that induce proper immunogenic tumor death can synergize with immune response modifiers to revolutionize cancer treatment [179]. Understanding the local and systemic immune mechanisms will lead to new potential therapeutic targets.

We predict that the future standard of care of lung cancer will involve patient tailored combination therapies that associate molecules that target specific genetic mutations or chemotherapeutic drugs with immune modulating agents, driven by the increasing understanding of the immune system in the cancer cell’s environment. The future for cancer treatment is bright if we are able to: I). Find a chemotherapeutic drug that induces immunogenic cell death in tumor cells while leaving the normal cells and stimulating immune cells intact. II). Explore ways to efficiently activate the good-natured immune system, for instance, the adoptive transfer of *in vitro* expanded activated T-cells or NK-cells, and III). Modulate the tumor environment to reduce local and systemic immune suppressive components while limiting potential side-effects for the patient; e.g. by the depletion of Treg by denileukin diftitox or polarizing the M2 macrophage towards the M1 subtype. The treatment has to be tuned to the cellular make-up of each patient individually, based on their own both tumoral and immunological characteristics, rather than by the anatomic location of the tumor in the body or by the tumor histology or genetic make-up. This individualized, multi-targeted approach will be able to redress the balance towards efficacious antitumor responses that can improve the overall survival for more patients.

## Abbreviations

APC: Antigen presenting cell(s); CTL: Cytotoxic T lymphocyte(s); CTLA-4: Cytotoxic T lymphocyte-associated antigen 4; DC: Dendritic cell(s); MDSC: Myeloid-derived suppressor cell(s); NK(T): Natural killer (T) cell(s); TAM: Tumor-associated macrophage(s); TIL: Tumor infiltration lymphocyte(s); Treg: Regulatory T cell(s).

## Competing interests

The authors declare that they have no competing interests.

## Authors’ contributions

MH contributed to literature research, data-analysis, interpretation of findings and drafting of the manuscript. JA contributed to study design, literature research, data-analysis, interpretation of findings and critical editing of the manuscript. RC contributed to literature research, data-analysis, interpretarion of findings and drafting of the manuscript. HG contributed to drafting of the manuscript. HH contributed to drafting of the manuscript. JH contributed to study design, literature research, data-analysis, interpretation of findings and critical editing of the manuscript. All authors read and approved of the final manuscript.

## Pre-publication history

The pre-publication history for this paper can be accessed here:

http://www.biomedcentral.com/1471-2407/12/580/prepub
